# Advancing Precision Diagnosis of Sarcopenic Obesity Through Digital Technologies, Wearables and Omics Data

**DOI:** 10.3390/life15121911

**Published:** 2025-12-13

**Authors:** Grigorios Panagiotou, Soren Brage

**Affiliations:** MRC Epidemiology Unit, Institute of Metabolic Science, University of Cambridge, Cambridge CB2 0QQ, UK

**Keywords:** precision diagnosis, sarcopenic obesity, sarcopenia, muscle loss, remote methodologies, eHealth, digital tools

## Abstract

Sarcopenic obesity, the coexistence of excess adiposity with loss of muscle mass and function, is becoming increasingly prevalent. The condition is linked to higher morbidity and mortality but its diagnosis remains limited by reliance on body composition methods that are costly, inaccessible, and/or involve radiation exposure. Recent advances in bioinformatics, data analytics, and digital health technologies create opportunities for scalable, precise approaches to detection. This narrative review synthesizes current evidence from the published literature on online medical libraries (Pubmed, Medline, Scopus, Google Scholar) until September 2025 on multi-omics, digital phenotyping and eHealth research, highlighting how these tools can refine risk stratification and extend diagnostic reach beyond traditional methods. We describe the potential utility of wearable sensor technologies, and smartphone-based body composition methods, as well as genomics, proteomics, transcriptomics and metabolomics. Such approaches, alone or in combination, may enable earlier identification of sarcopenic obesity, including in individuals who are not routinely prioritized for screening. We conclude that integrating biological and digital data offers promise for advancing precision diagnostics in sarcopenic obesity, enabling more tailored prevention and intervention strategies while ultimately reducing healthcare burden. Further research is required to determine the feasibility, clinical utility and scalability of such innovations before their widespread implementation.

## 1. Introduction

The global obesity epidemic represents a major healthcare and socioeconomic challenge, contributing significantly to cardiovascular and metabolic mortality [1]. The high rates of obesity are driving the increase in related comorbidities, including cardiovascular disease [2], type 2 diabetes [3] and cancer [4,5], that results in both personal suffering and substantial burden on the healthcare system. Treatment of obesity-related conditions has an estimated annual expenditure of $190 billion in the United States alone [6]. The global economic impact is projected to surpass $4.3 trillion annually in the next 10 years, reaching 3% of the global gross domestic product (GDP), which is comparable to the COVID-19 pandemic [7]. At the same time, such investments as well as other pharmacological advances in chronic disease management, including obesity-related comorbidities, have increased life expectancy, unveiling new age-related challenges [8]. While body mass index (BMI) tends to plateau in old age, waist circumference (WC) often continues to rise, reflecting progressive visceral adiposity alongside declines in fat-free mass, particularly muscle and bone [9].

Age-related muscle loss begins in the fourth decade of life, progressing at 3–8% per decade and accelerating after the eighth decade to nearly 1% annually in men and 0.7% in women. Muscle strength declines even faster, at up to 4% and 3% per year in men and women, respectively [3,4]. This accelerated decline, termed sarcopenia [10] remains without FDA recognition as a treatable condition despite emerging therapeutic strategies [11].

Sarcopenic obesity (SO), defined as the coexistence of excess adiposity together with low muscle mass and function, exacerbates metabolic, functional, and psychological complications beyond those of either sarcopenia or obesity alone [9,12,13,14]. Its global prevalence is estimated at approximately 11% among adults aged ≥ 60 years [15], though SO may occur at any age, prompting the alternative term “obesity with low lean muscle mass” [16]. Individuals diagnosed with the condition are at higher risk of falls and fractures [17,18,19], frailty and disability [20,21,22,23], as well as reduced quality of life and adverse health outcomes [24,25]. SO is also associated with dysglycaemia [26,27,28], risk of malignancies [29] and adverse cancer-related outcomes [30,31], and increased cardiovascular and all-cause mortality [32,33], more than sarcopenia or obesity alone, thereby constituting a significant public health concern.

SO arises through multiple interacting pathways, including protein degradation rather than lack of protein synthesis [34], age-related hormonal decline [35], such as a reduction in sex and adrenal steroids [36,37], growth hormone [38] and vitamin D deficiency [39]. Many studies have shown the role of intra-muscular inflammation [40] and adipose tissue infiltration, as well as an increase in intramyocellular lipid accumulation causing lipotoxiticy [41], and other muscle-specific alterations including altered myokine signalling [42,43] and cellular senescence [44,45]. Gut dysbiosis [46] and other behavioural factors such as Western diet, and physical inactivity, as well as chronodistruption and disturbed sleep patterns [47], further contribute to its development. Environmental and socioeconomic factors, such as lower educational attainment, have also been linked with higher odds of developing SO [48].

There is an urgent need for better diagnostics for SO. Given its heterogeneity, precision approaches integrating digital health tools, multi-omics and bioinformatics may enable earlier diagnosis and targeted treatment or prevention. In the present review, we synthesize the evidence on the potential benefits of introducing wearable biosensor methodologies in tandem with genomics, proteomics, transcriptomics and metabolomics for the timely diagnosis of SO that may inform the design of tailored, scalable prevention strategies across different age and ethnic groups in the future.

## 2. Review Methodology

This narrative review synthesizes current evidence on SO as a model for precision diagnosis in the cardiometabolic field. A search for human studies published in the English language in online medical libraries (PubMed, Medline, Scopus, Google Scholar) was conducted through September 2025 using keywords including “sarcopenia”; “sarcopenic obesity”; “precision diagnosis”; “multi-omics”; “bioinformatics”; “digital tools”, “muscle decline”, “myopenia/dynapenia”, “wearables”, “biosensors”, “artificial intelligence”, “machine learning”, “eHealth”, “genomics”, “proteomics”, “transcriptomics” and “metabolomics”. Exclusion criteria were articles not written in English, published in journals that are not indexed on Medline and referring to SO secondary to endocrine diseases. Due to population and study design heterogeneity, a systematic review was not feasible; thus, a narrative synthesis was adopted. In the following sections, we first outline current definitions and diagnostic challenges of SO, then review evidence from multi-omics and big-data studies, and finally discuss the role of digital health technologies in advancing precision diagnosis. We conclude by highlighting knowledge gaps and opportunities for integration into clinical practice.

## 3. Diagnosis and Screening

Historically, a major limitation in SO research has been the lack of uniform diagnostic criteria [49]. Recently, a joint statement by the European Association for the Study of Obesity and the European Society for Clinical Nutrition and Metabolism proposed an algorithm for SO diagnosis [50]. Similar criteria have been introduced by The Asia–Oceania Association for the Study of Obesity (AOASO) and the International Association of Gerontology and Geriatrics Asia/Oceania Region (IAGG-AOR), with cutoffs tailored to Asia-Oceania populations [51].

Diagnosis follows a two-step process, followed by a stage classification:

Assessment of muscle function: Before proceeding with imaging studies, the proposed algorithm recommends functional assessments for dynapenia (muscle dysfunction), using hand dynamometer for handgrip strength, knee extensor strength and/or physical performance tests, such as the 30-s chair-stand test and/or the 5-times sit-to-stand test [50].

Quantification of muscle mass: For those with confirmed dynapenia, body composition can be measured via dual-energy X-ray absorptiometry (DEXA) or, where unavailable, bioelectrical impedance analysis (BIA). Computed tomography (CT) may be used opportunistically for patients undergoing scans for other clinical reasons. Ethnicity-specific cut-offs and reference ranges should be applied [50].

Once diagnosed, SO is staged based on the presence of complications: Stage I indicates the absence of SO-related complications, while Stage II denotes their presence [50].

Screening, on the other hand, should focus on at-risk individuals, specifically those with obesity (based on BMI and/or waist circumference) presenting symptoms or risk factors for sarcopenia, with or without the aid of validated self-report questionnaires such as the SARC-F a five-question screening tool for sarcopenia, with questions on Strength, Assistance with walking, Rise from a chair, Climb stairs, and Falls, to assess physical function [52,53]. Despite advocating an obesity-dependent stepwise approach, current practices primarily target adults aged ≥70 years, when sarcopenia classically presents, with overweight or obesity, or those with comorbidities contributing to muscle loss [50]. This may be overly restrictive, as SO can develop in younger individuals and often presents with non-specific symptoms that overlap with other conditions. Indeed, the AOASO/IAG-AOR consensus proposes initiating screening at the age of 40 years [51].

However, screening tools such as the SARC-F have not been validated in younger populations and may therefore be inadequate for early prevention. Additionally, BMI is an imperfect proxy for body composition and adiposity and should primarily be used in individuals with BMI > 40 kg/m^2^ [54]. Exclusive reliance on BMI for obesity assessment risks overlooking individuals with higher levels of adiposity and concomitant myopenia who remain within the normal-weight range. Early body composition assessment is often omitted in proposed screening algorithms [50], thus potentially missing individuals with modest BMI or waist circumference but elevated adiposity. Moreover, while sarcopenia screening tools demonstrate good specificity, they have limited sensitivity [55] which hinders early identification of cases and results in missed opportunities for efficient preventive programs.

Emerging approaches including genetic or biochemical biomarkers combined with behavioural and clinical parameters may enhance early detection, though progress in biomarker development for sarcopenia has been historically challenging [56]. Identifying at-risk individuals before the onset of clinically significant disease is critical to preventing SO and may enable targeted risk modification in younger populations. “Pre-sarcopenia” can potentially be detected using non-invasive methods such as BIA and handgrip strength in routine clinical settings [57]. However, currently early detection remains heavily reliant on clinical awareness, leaving otherwise healthy, community-dwelling individuals with subtle symptoms at risk of being overlooked. Thus, implementing scalable and accessible screening approaches, such as combining biomarker testing and digital health (eHealth) methodologies, may enhance the diagnostic capacity of current strategies and facilitate early identification of at-risk individuals.

Given the complexity and heterogeneity of SO, stratifying patients based on shared clinical and behavioural characteristics may enhance the efficacy of prevention and treatment strategies, and lead to precision interventions tailored to individual patient needs. However, until the feasibility and efficacy of such approaches are tested clinically, personalised interventions should complement, rather than replace, established therapeutic treatment pathways [58]. Moreover, early recognition through targeted screening is central to prevention, yet no formal guidelines currently exist for population-based risk stratification or precision prevention. Clinicians must therefore rely on accessible diagnostic tools, including muscle strength and body composition assessments, for individuals at risk once clinical suspicion arises.

Furthermore, with the growing recognition of SO as a distinct therapeutic target, emerging evidence highlights potential risks associated with “one-size-fits-all” weight-loss strategies in this population [59,60]. Current clinical guidelines recommend that weight-reducing interventions in older adults should only be pursued following a careful, individualised assessment of both risks and anticipated benefits [60]. When weight loss is deemed appropriate, a balanced dietary approach is advised, including moderate caloric restriction (~500 kcal/day below estimated requirements), sufficient high-quality protein intake (~1.2 g/kg body weight per day, ideally distributed as 25–30 g per meal) [61], and adequate intake of essential micronutrients such as vitamin D, vitamin C, and calcium [62]. Given the heightened risk of bone and muscle loss in this group, careful monitoring is essential. Weight reduction should be gradual, targeting 0.25–1 kg per week, and combined with an exercise program to preserve lean mass [60].

## 4. Digital Tools and At-Home Testing

While a variety of digital tools, including wearables, sensors, smart-scales and smartphone applications, have been used to complement and/or augment existing treatment strategies in SO [63], the diagnostic potential of such tools is less explored.

The increasing use of artificial intelligence analytics, including machine learning, along with the expansion of wearable devices capturing real-life data, has enabled the development of predictive algorithms for the detection of SO. In a study evaluating physical fitness data from older Korean adults, a neural network model detected SO with a validation accuracy of 93.1%, based on body fat percentage assessed by BIA and grip strength data [64]. Zambon Azevedo et al. developed an unbiased diagnostic approach for SO using unsupervised machine learning algorithms based on DXA-derived body composition data from a cross-sectional study in the French population (n = 1427) [65]. In another study, a web application was developed using an 8-feature model including demographic, anthropometric, and physical performance data for the prediction of SO [66].

Utilizing objectively measured physical activity or gait-based data from accelerometers and other wearable devices for SO detection at a population level may further increase the probability of diagnosis SO at-scale, followed by the prescription of individualized digital interventions.

Several studies have demonstrated a positive effect of eHealth interventions on health promotion [67,68,69]. Schoufour et al. suggested that peri-retirement is an ideal age window to target individuals at higher risk of developing SO and to promote diet and exercise changes using digital health technologies [70]. A recent feasibility study successfully delivered a remote intervention to improve sarcopenia through tailored feedback and wearable data in 60 older adults with a mean age of 74 years and mild frailty [71]. Digital illiteracy is gradually decreasing, and such technologies are generally well accepted in this age group [72]. These methods, including smartphone applications and wearables, offer the additional benefit of at-home interventions, reducing overall healthcare costs and shifting care away from secondary settings.

Regarding body composition assessment, DEXA remains the most commonly used clinical imaging standard including whole-body or segmental measurements, while other methods such as CT, MRI, and ultrasound have also been used in clinical settings [73]. BIA is a more accessible alternative but is less accurate [74]. Recent innovations have enabled novel approaches to assess muscle health [75]. For example, advances in imaging technology have led to development of three-dimensional optical imaging for body composition through body shape using smartphone apps [76,77,78] including bone mineral content, with high accuracy [79], a critical component of SO pathophysiology given the increased fracture risk in those patients [17,80]. Smartwatch-based BIA methodologies have also been developed [81,82,83]. Moreover, a video analysis application using the sit-to-stand test for sarcopenia detection demonstrated good diagnostic performance in community-dwelling older adults, allowing remote evaluation of muscle function alongside body composition measurements [84]. These innovations enable inexpensive and non-invasive longitudinal assessments of lean and bone mass. The on-demand attribute of these methods is particularly relevant in individuals experiencing rapid body composition changes, such as those prescribed weight-loss medications, improving treatment safety through preventing iatrogenic sarcopenia and/or supporting individualized therapeutic decisions, such as encouraging increased protein intake and/or resistance exercise, and promoting patient autonomy through real-time biofeedback. They may also be applied in other muscle disorders, including cachexia, neuromuscular diseases, or genetic syndromes affecting body composition, such as lipodystrophy syndromes. However, the feasibility, validity, and reliability of these methods should first be evaluated in high-risk groups before application to individuals with extreme body composition phenotypes, including those with SO.

Furthermore, machine-learning methodologies combined with widely available wearable devices could inform predictive or surveillance models in at-risk individuals based on free-living data. While these approaches are increasingly used for wellness and lifestyle applications, their use in detecting altered body phenotypes is understudied. Wearable sensor data have been used to evaluate frailty in community-dwelling older adults [85]. Several studies have examined associations between wearable and accelerometer-derived data, including gait monitoring parameters, and indices of sarcopenia and frailty [86,87,88,89]. As accelerometers are very common sensors in wearables, these findings highlight the potential of such technologies to serve as the basis for future detection algorithms and early screening tools for SO. A meta-analysis of seven studies concluded that more advanced movement sensors such as inertial measurement units (which in addition to an accelerometer, also includes a gyroscope and a magnetometer) combined with artificial intelligence models could be an effective tool for sarcopenia screening based on gait analysis [90]. Measuring dynamic plantar pressure with a flexible printed piezoelectric sensor array connected via Bluetooth to a smartphone demonstrated over 93% accuracy for sarcopenia detection in 51 individuals aged 55 years and above [91]. A similar fabric-based piezoresistive sensing system applied to the soles of the feet identified walking speed and gait abnormalities for sarcopenia detection and could potentially monitor other body regions or smaller muscles, such as analysis of voice for vocal muscles, as well as heart rate detection [92]. Other approaches include wearable surface electromyography incorporated into socks [93] or signals from stimulated muscle contraction [94]. We have also recently reviewed the potential applications of continuous glucose monitoring (CGM) technologies in people at-risk of diabetes, including those with SO [95], highlighting two studies that used CGM to derive algorithms for postprandial impaired glucose tolerance and/or muscle insulin resistance [96,97], a trait of the declining muscle in SO, thereby allowing for early identification of individuals with dysglycaemia before the development of clinical diabetes [95]. These potential use cases of digital tools in SO are summarized in Table 1.

Whether the diagnostic accuracy of these methodologies is affected by adiposity or bone loss remains to be determined. It is important to note that so far these technologies have been predominantly tested in the general population, largely in people of White-Caucasian ethnic background. Therefore, further validation is needed in people with obesity and other ethnic groups, such as Asian populations with different BMI cut-offs for obesity, as well as in younger and pediatric populations. If proven robust, such technologies could enable earlier, non-invasive, and out-of-hospital detection and risk stratification of individuals with SO, and may be incorporated into community-based programs to monitor therapeutic efficacy. However, before their adoption in routine clinical practice, diagnostic validity, reproducibility, and cost-effectiveness must be confirmed in large-scale longitudinal studies and randomized controlled trials. Furthermore, as smartphone-based body composition tools rely on body shape and surface imaging, potential limitations should be carefully evaluated in patient populations commonly associated with SO and abnormal body composition, including those with peripheral edema, heart failure, liver or kidney disease, and/or in pregnancy. Edema and peripheral fluid accumulation can increase tissue volume leading to systematic overestimation of adiposity and potentially underestimation of lean mass when relying on external morphology alone [98].

## 5. Multi-Omics

An essential step toward understanding the complex pathophysiology of SO and achieving precision prevention and treatment is the identification of targeted pathophysiological pathways and biomarkers using novel multi-omics approaches. While such methodologies have been applied in preclinical models of SO and in studies of patients with either obesity [99,100,101] or sarcopenia alone [102,103], evidence specifically addressing patients with a coexisting SO phenotype remains limited. Given its multifactorial nature, multiple mechanistic pathways likely contribute to SO, with certain genes and pathways implicated in either sarcopenia or obesity alone potentially also influencing susceptibility [104]. In this review we focused on multi-omics studies evaluating the co-existing phenotype of SO in humans, including genomics, transcriptomics, proteomics and metabolomics analyses.

A previous genome-wide association study (GWAS) showed that *FTO* gene, which is classically associated with obesity, is also associated with lean mass [105], while a more recent GWAS using UK Biobank data has identified 78 single nucleotide polymorphisms associated with sarcopenia markers, of which 55 were also linked to body fat percentage and adiposity, suggesting these loci may serve as genomic predictors of SO [106]. This condition is primarily associated with aging, and therefore less likely to have a strong genomic basis; however, epigenomic changes arising from cumulative cellular damage may still influence the development of SO, although supportive evidence is still missing.

Expanding on these findings, Xu et al. [107] applied exome-wide sequencing using UK Biobank data and identified a common variant in the long noncoding RNA *LYPLAL1 Antisense RNA 1* (*LYPLAL1-AS1*), a gene implicated in adipocyte differentiation in preclinical models [108], as significantly associated with SO. Co-localization analysis supported this association, indicating a potential role for *LYPLAL1 AS1* in the pathogenesis of SO. This study also highlighted adiponectin as a protein co-localized with *LYPLAL1 AS1*, suggesting interplay between these molecules in disease development [107]. Rare variants in five additional genes, including phosphodiesterase 3B (*PDE3B*), myozesin 3 (*MYOZ3*), solute carrier family 15 member 3 (*SLC15A3*), ring finger protein 130 (*RNF130*), and tyrosine kinase non-receptor 2 (*TNK2*), were also associated with the SO phenotype in that study [107]. Further supporting these findings, single nucleotide polymorphisms in the resistin (*RETN*) gene were linked to a SO index defined by increased visceral fat area and reduced muscle grip strength [109].

Transcriptomic analyses have provided additional insight. Obesity and adiposity are associated with a distinctive transcriptomic profile of skeletal muscle in elderly individuals, including genes and pathways related to DNA methylation, inflammation and longevity [110]. Using artificial neural network inference, Tarum et al. examined aging muscle transcriptomes from RNA sequencing datasets in young and older individuals from the Gene Expression Omnibus repository [111]. This approach identified complex gene interactions and pathways involved in muscle aging. Validation with real-time quantitative PCR in muscle biopsies confirmed differential expression of genes including Ubiquitin Specific Peptidase 54 (*USP54*), Chondroadherin (*CHAD*) and Zinc Finger DBF-Type containing 2 (*ZDBF2*), which were significantly upregulated in older adults [111]. Gene ontology analysis further highlighted the involvement of apoptotic, immune response, and bone development pathways in age related muscle decline [111].

A detailed transcriptome network encompassing pathways related to cell turnover, protein and muscle synthesis, proteolysis, muscle contraction, lipid and nutrient metabolism, and hormone signaling was also identified in hormone-related sarcopenia models of ovariectomized rats with osteosarcopenic obesity [112]. Similarly, human studies integrating proteomic and transcriptomic approaches have characterized molecular interactions in sarcopenia and osteoporosis, identifying genes involved in osteoclast differentiation and cytokine–cytokine receptor interaction, including Protein Disulfide Isomerase family A member 5 (*PDIA5*), Tubulin Beta 1 Class VI (*TUBB1*), Cytoplasmic FMR1-interacting protein 2 (*CYFIP2*), Myosin Heavy Chain 7 (*MYH7*), and Neural cell adhesion molecule 1 (*NCAM1*) [113]. Furthermore, using integrated transcriptomics, Yu et al. recently identified a set of 208 common differentially expressed genes (CDEGs) that were significantly enriched in mitochondrial oxidative phosphorylation pathways. Among these, four key genes, including Succinate Dehydrogenase Complex Iron Sulfur Subunit B (*SDHB*), Succinate Dehydrogenase Complex Subunit D (*SDHD*), ATP Synthase F1 Subunit Alpha (*ATP5F1A*), and ATP Synthase F1 Subunit Beta (*ATP5F1B*), were downregulated and represent core components of mitochondrial respiratory chain complexes suggesting that impaired mitochondrial function constitutes a shared pathological mechanism linking sarcopenia and obesity [114].

Proteomic studies in SO remain limited. In a prospective study with a median follow up of 13.5 years, C-C motif chemokine 28 and metalloproteinase inhibitor 4 were associated with concurrent low muscle mass and high fat mass cross-sectionally, while N-terminal prohormone brain natriuretic peptide (NT-proBNP) was linked to longitudinal muscle mass loss and fat mass gain [115]. These findings suggest a potential mechanistic link between SO and heart failure, consistent with the elevated cardiovascular risk observed in patients.

Indeed, Jia et al. confirmed that the relationship between SO and heart failure was mediated by a metabolomic risk score comprising 12 metabolites in 22,500 UK Biobank participants with type 2 diabetes [115]. Complementary approaches using ^31^-phosphorus magnetic resonance spectroscopy, metabolomics, and lipidomics in muscle biopsies identified phosphorus metabolites including phosphocreatine and phosphodiesterase 2 (PDE2) as markers of muscle health decline in older adults [116]. Additional untargeted studies have further expanded the catalogue of potential biomarkers implicated in aging-related muscle loss [117,118]. Collectively, these studies underscore the potential of genetic and molecular markers to elucidate the underlying biology and identify novel intervention targets. Table 2 summarizes the omics research in SO.

## 6. Discussion and Direction of Future Research

Digital phenotyping and multi-omics biomarkers differ in scale, format and biological meaning. However, these modalities could be integrated within unified analytical frameworks to provide a more comprehensive, multidimensional characterization of disease risk. Currently, such methodologies have not entered routine clinical practice and the feasibility of those approaches, as well as the cost-effectiveness, should be examined in future studies. Importantly, each modality captures a distinct but complementary layer of risk characterization contributing to an individualized profile. Data from wearables and smartphone apps provide continuous, real-world information on physical activity, gait characteristics, eating and sleep habits and easy-to-capture, at-home body composition measures. On the other hand, multi-omics data provide insights on biological susceptibility and molecular pathways involved in SO pathophysiology. Integrating those heterogeneous data sources through data harmonization into advanced statistical inference models can increase their predictive performance. Due to their complementarity and interaction, combining multiple measurement modalities has the potential to outperform single-modality strategies and increased precision at the individual level, thereby leading to the development of tailored prevention and treatment algorithms. However, such algorithms have not been developed yet, and their clinical utility should be tested in the future. In the meantime, while detailed individualization or personalization of care through bioinformatics and machine learning methodologies may not yet be fully achievable due to current technical and practical limitations, a precision approach using routinely obtained clinical, biochemical, demographic and behavioral parameters remains highly desirable given the complexity and inter-individual variability of SO.

The translation of omics biomarkers from research settings into clinical practice requires a structured pathway encompassing analytical validation, demonstration of reproducibility across platforms and populations, clinical validation to establish relevance and predictive value, as well as assessment of feasibility and health economic considerations to support future large-scale implementation. In practice, precision strategies should initially serve as supportive tools that augment established diagnostic approaches until their long-term predictive and clinical utility is confirmed in longitudinal studies. As these technologies continue to develop, their performance should be rigorously compared with conventional strategies in head-to-head clinical trials. Nevertheless, while the integration of such techniques into routine practice is still evolving, the holistic management of patients with SO aimed at reducing frailty, functional dependency, and adverse cardiometabolic outcomes remains an urgent priority.

Building on the laboratory and technological advancements described above, we advocate a multifaceted, precision-informed approach for the early identification and diagnosis of individuals at risk of or with established SO. This approach should integrate individual demographics, lifestyle behaviors, biochemical and hormonal profiles, digital technologies including wearable sensors, and smartphone or eHealth data, remote body composition assessments, relevant clinical parameters, and eventually multi-omics analyses. Large, deeply phenotyped cohorts combining body composition, wearable, and bioinformatics data are essential to determine the clinical utility of these modalities when applied in combination for SO detection.

Such strategies could enable large-scale, remote screening without requiring hospital or clinic visits, while also informing individualized profiles to guide targeted interventions in exercise, nutrition, and precision medicine. By leveraging digital tools, and multi-omics approaches, this framework has the potential to substantially improve diagnostic accuracy, early detection, and personalized management in SO. However, until these tools are more thoroughly validated, clinicians should employ subgroup stratification to facilitate more precise and targeted interventions for individuals at risk of or with SO.

Future research studies should focus on validating these digital methodologies as diagnostic tools across diverse populations and clinical contexts, and on assessing their clinical utility in supporting personalized treatment strategies that enhance patient autonomy through real-time biofeedback. Parallel studies to evaluate the clinical validity and predictive value of multi-omics markers in SO are also needed, as these will inform health economic analyses and guide the future integration of such technologies into routine clinical practice.

## 7. Conclusions

SO represents a complex and increasingly prevalent condition with significant comorbidities, yet current diagnostics for SO remain limited, often inaccessible and primarily focused on older populations. Emerging advances in digital health methodologies and bioinformatics offer new opportunities for scalable, precision diagnostic strategies. Integrating biological and digital data offers promise for advancing precision diagnostics in SO, enabling more tailored prevention and intervention strategies while potentially reducing healthcare burden. A precision diagnostic framework combining biological, functional and behavioral markers may ultimately shift SO management toward more proactive, personalized, and equitable care. Nevertheless, further research is required to determine the feasibility, clinical utility and scalability of such innovations before their widespread implementation.

## Figures and Tables

**Table 1:** Emerging digital health tools and artificial intelligence (AI) applications with potential for early detection, monitoring, and personalized management of sarcopenic obesity (SO).

Technology/Approach	Key Findings/Features	Study Design/Sample Size	Implications for SO	Drawbacks/Limitations
Body fat percentage (via BIA) and grip strength Artificial intelligence (AI) and neural network models	Neural network model achieved 93.1% validation accuracy for SO detection	Large population cocohort (n = 107,545) of older Korean adults [64]	Demonstrates the feasibility of AI in accurately identifying SO based on simple clinical and body composition parameters	Accuracy dependent on quality of BIA measurements.
Unsupervised machine learning algorithms based on DEXA	Diagnostic model for SO and associated comorbidities	Large cross-sectional study of people with overweight/obesity (n = 1165), validation dataset (n = 262), French population [65]	Enables unbiased classification and stratification of SO risk phenotypes	DEXA not scalable for population screening. Limited generalizability in ethnically and clinically diverse populations.
Web-based predictive models	Web application using an 8-feature model (demographic, anthropometric, and physical performance data) predicted SO	Web-based predictive tool, derivation cohort (n = 1431), validation cohort (n = 832) [66]	Offers a scalable and accessible platform for community-level SO screening	External validation and calibration needed before adoption across different healthcare settings.
Smartphone-based imaging and 3D optical body composition assessment	3D imaging, BIA and body-shape analysis using smartphones provide accurate estimates of body composition and bone mineral content	Imaging validation studies [75,76,77,78,79,81,82,83]	Enables non-invasive, low-cost, and remote assessment of lean and bone mass relevant to SO	Limited validation in clinical populations. Accuracy may be affected by fluid retention. Not validated in altered body composition phenotypes, pregnancy and pediatric populations. Algorithm may not perform uniformly across ethnic groups.
Wearables and accelerometer-based models	Wearable sensor data used to evaluate frailty and sarcopenia indices; AI gait analysis via inertial measurement units effective in screening	Community-dwelling older adults [85,86,87,88,89,90]	Highlights the role of wearables in early detection and continuous monitoring of SO	Lack of standardized data formats and sensor specifications limits harmonization. Device-specific algorithms reduce comparability.
Video-based functional testing	Sit-to-stand video analysis app demonstrated good diagnostic performance for sarcopenia detection	Cohort study of community-dwelling older Spanish adults (n = 686) [84]	Facilitates remote muscle function assessment complementing body composition metrics	Performance depends on camera quality and user compliance. Requires digital literacy.
Wearable piezoelectric and piezoresistive sensors	Flexible piezoelectric plantar sensors achieved >93% accuracy in detecting sarcopenia; fabric-based sensors tracked gait and mobility	Small case–control (sarcopenia vs. control) sensor-based detection studies [91,92]	Potential for real-time mobility tracking and SO screening through gait and balance monitoring	Lack of validation in SO and elderly populations limits generalizability of the results. Unclear performance in patients with foot deformities and peripheral neuropathy.
Wearable electromyography and muscle contraction sensors	Surface EMG integrated into socks and muscle stimulation signal analysis proposed for sarcopenia monitoring	Prototype sensor development. Healthy volunteers (n = 5) [93] & case–control (sarcopenia vs. non-sarcopenia) design (n = 199) [94]	Enables non-invasive muscle performance tracking and potential integration into digital SO monitoring frameworks	Unclear long-term usability. Lack of validation in elderly population.
Integrated Continuous Glucose Monitoring and multimodal wearable data	High predictive accuracy (AUC up to 95%) in detecting muscle insulin resistance and metabolic heterogeneity, highlighting the role of circadian and behavioral patterns in metabolic health.	Small cohort studies, n = 32 [96] n = 36 [97]	Combining continuous biosensor data with lifestyle monitoring may be able to capture early metabolic alterations relevant to SO, such as muscle insulin resistance, supporting precision digital screening and phenotyping of individuals at risk.	Increased cost, lack of defined criteria for muscle insulin resistance and validation in non-diabetic populations.
eHealth and digital health interventions	Remote programs based on wearable data and tailored feedback improved sarcopenia	Feasibility two-arm RCT, arms: tailored feedback on sitting, standing and stepping, health coaching and wearables vs. control (n = 60, mean age 74 years; duration 6 months) [78]	Supports delivery of lifestyle modification through remote, personalized interventions	Limited sample size and short follow-up. Findings may vary with adherence. Not yet validated in individuals with SO.

Abbreviations: AI, artificial intelligence; BIA, bioelectrical impedance analysis; DEXA, Dual X-ray absortiometry; EMG, electromyography; RCT, randomised controlled trial; SO, sarcopenic obesity.

**Table 2:** Multi-omics approaches in sarcopenic obesity (SO).

Omics Type	Key Findings/Biomarkers	Study Design/Model	Implications for SO	Drawbacks/Limitations	References
Genomics	78 single nucleotide polymorphisms associated with sarcopenia markers, 55 overlapping with adiposity; rare variants in *PDE3B*, *MYOZ3*, *SLC15A3*, *RNF130*, *TNK2*; *RETN* variants associated with SO index	UK Biobank, exome and genome-wide association studies	Genetic predictors of susceptibility to SO and shared genomic loci between sarcopenia and obesity	Genomic risk does not capture environmental drivers of SO.	[105,106,107,108,109]
Long noncoding RNA	*LYPLAL1-AS1* variant associated with SO; co-localized with adiponectin	UK Biobank, exome-wide sequencing	Possible regulatory role in adipocyte differentiation and SO pathogenesis	Requires replication across different cohorts.	[107,108]
Transcriptomics	Upregulation of *USP54*, *CHAD*, *ZDBF2* in aging muscle; pathways include apoptosis, immune response, bone development	Human RNA-sequencing datasets, artificial neural network inference	Insight into molecular pathways contributing to muscle aging and SO	Tissue-specific effects, muscle biopsies are invasive procedures and not practical in routine care.	[110,111]
	208 common differentially expressed genes enriched in mitochondrial oxidative phosphorylation; downregulation of *SDHB*, *SDHD ATP5F1A*, *ATP5F1B*	Human integrated transcriptomic analyses	Mitochondrial dysfunction as a shared pathological mechanism linking sarcopenia and obesity	Mitochondrial dysfunction may reflect aging or comorbidities rather than SO specifically.	[114]
Proteomics	*PDIA5*, *TUBB1*, *CYFIP2*, *MYH7*, *NCAM1* involved in muscle and bone pathways	Human proteomic and transcriptomic integration studies	Candidate biomarkers and therapeutic targets linking muscle and bone metabolism	Variability with inflammation, comorbid disease and across platforms limits comparability.	[113]
	C-C motif chemokine 28, metalloproteinase inhibitor 4, and NT-proBNP associated with concurrent low muscle mass and high fat mass and longitudinal body composition change	Longitudinal cohort, median 13.5-year follow-up	Potential biomarkers connecting SO to heart failure and cardiometabolic complications	Requires replication and further validation in SO.	[115]
Metabolomics and lipidomics	Phosphocreatine, PDE2, and multiple phospholipids identified as markers of muscle health; 12-metabolite risk score mediating SO–heart failure relationship	UK Biobank, 31-phosphorus magnetic resonance spectroscopy, muscle biopsies	Biomarkers linking metabolic dysregulation, muscle decline, and cardiovascular risk in SO	Metabolite concentrations may be influenced by behaviors. Lack of standardization across laboratories and absence of defined reference range for clinical use.	[116,117,118]

Abbreviations: *PDE3B*, phosphodiesterase 3B; *MYOZ3*, myozesin 3; *SLC15A3*, solute carrier family 15 member 3; *RNF130*, ring finger protein 130; *TNK2*, tyrosine kinase non-receptor 2; *RETN,* resistin gene; *LYPLAL1-AS1*, long noncoding RNA *LYPLAL1 Antisense RNA 1*; *USP54*, Ubiquitin Specific Peptidase 54; *CHAD*, Chondroadherin; *SDHB*, succinate dehydrogenase complex iron sulfur subunit B; *SDHD*, succinate dehydrogenase complex subunit D; *ATP5F1A*, ATP synthase F1 subunit alpha; *ATP5F1B*, ATP synthase F1 subunit beta; *ZDBF2*, Zinc Finger DBF-Type containing 2; *PDIA5,* Protein Disulfide Isomerase family A member 5; *TUBB1*, Tubulin Beta 1 Class VI; *CYFIP2*, Cytoplasmic FMR1-interacting protein 2; *MYH7*, Myosin Heavy Chain 7; *NCAM1*, Neural cell adhesion molecule 1; NT-proBNP, N-terminal prohormone brain natriuretic peptide, PDE2, phosphodiesterase 2.

## Data Availability

No new data were created or analyzed in this study.

## References

[1] Afshin A., Forouzanfar M.H., Reitsma M.B., Sur P., Estep K., Lee A., Marczak L., Mokdad A.H., Moradi-Lakeh M., GBD 2015 Obesity Collaborators (2017). Health Effects of Overweight and Obesity in 195 Countries over 25 Years. N. Engl. J. Med..

[2] Twig G., Yaniv G., Levine H., Leiba A., Goldberger N., Derazne E., Shor D.B.-A., Tzur D., Afek A., Shamiss A. (2016). Body-Mass Index in 2.3 Million Adolescents and Cardiovascular Death in Adulthood. N. Engl. J. Med..

[3] Bjerregaard L.G., Jensen B.W., Ängquist L., Osler M., Sørensen T.I.A., Baker J.L. (2018). Change in Overweight from Childhood to Early Adulthood and Risk of Type 2 Diabetes. N. Engl. J. Med..

[4] Calle E.E., Rodriguez C., Walker-Thurmond K., Thun M.J. (2003). Overweight, Obesity, and Mortality from Cancer in a Prospectively Studied Cohort of U.S. Adults. N. Engl. J. Med..

[5] Arnold M., Pandeya N., Byrnes G., Renehan A.G., Stevens G.A., Ezzati M., Ferlay J., Miranda J.J., Romieu I., Dikshit R. (2015). Global Burden of Cancer Attributable to High Body-Mass Index in 2012: A Population-Based Study. Lancet Oncol..

[6] Cawley J., Meyerhoefer C. (2012). The Medical Care Costs of Obesity: An Instrumental Variables Approach. J. Health Econ..

[7] Economic Impact of Overweight and Obesity to Surpass $4 Trillion by 2035. World Obesity Federation. https://www.worldobesity.org/news/economic-impact-of-overweight-and-obesity-to-surpass-4-trillion-by-2035.

[8] Sattar N., McMurray J.J.V., McInnes I.B., Aroda V.R., Lean M.E.J. (2023). Treating Chronic Diseases without Tackling Excess Adiposity Promotes Multimorbidity. Lancet Diabetes Endocrinol..

[9] Atkins J.L., Wannamathee S.G. (2020). Sarcopenic Obesity in Ageing: Cardiovascular Outcomes and Mortality. Br. J. Nutr..

[10] Cruz-Jentoft A.J., Sayer A.A. (2019). Sarcopenia. Lancet.

[11] Evans W.J., Guralnik J., Cawthon P., Appleby J., Landi F., Clarke L., Vellas B., Ferrucci L., Roubenoff R. (2023). Sarcopenia: No Consensus, No Diagnostic Criteria, and No Approved Indication—How Did We Get Here?. Geroscience.

[12] Wannamethee S.G., Atkins J.L. (2015). Muscle Loss and Obesity: The Health Implications of Sarcopenia and Sarcopenic Obesity. Proc. Nutr. Soc..

[13] Batsis J.A., Villareal D.T. (2018). Sarcopenic Obesity in Older Adults: Aetiology, Epidemiology and Treatment Strategies. Nat. Rev. Endocrinol..

[14] Prado C.M., Batsis J.A., Donini L.M., Gonzalez M.C., Siervo M. (2024). Sarcopenic Obesity in Older Adults: A Clinical Overview. Nat. Rev. Endocrinol..

[15] Gao Q., Mei F., Shang Y., Hu K., Chen F., Zhao L., Ma B. (2021). Global Prevalence of Sarcopenic Obesity in Older Adults: A Systematic Review and Meta-Analysis. Clin. Nutr..

[16] Murdock D.J., Wu N., Grimsby J.S., Calle R.A., Donahue S., Glass D.J., Sleeman M.W., Sanchez R.J. (2022). The Prevalence of Low Muscle Mass Associated with Obesity in the USA. Skelet. Muscle.

[17] Yeung S.S.Y., Reijnierse E.M., Pham V.K., Trappenburg M.C., Lim W.K., Meskers C.G.M., Maier A.B. (2019). Sarcopenia and Its Association with Falls and Fractures in Older Adults: A Systematic Review and Meta-analysis. J. Cachexia Sarcopenia Muscle.

[18] Wen Q., Chen X., Luo S., Hou L., Yue J., Liu X., Xia X., Liu F., Dong B., Ge N. (2024). Association between Sarcopenia Grade and Fall History among Older Adults in West China: A Retrospective Study. BMJ Open.

[19] Landi F., Liperoti R., Russo A., Giovannini S., Tosato M., Capoluongo E., Bernabei R., Onder G. (2012). Sarcopenia as a Risk Factor for Falls in Elderly Individuals: Results from the IlSIRENTE Study. Clin. Nutr..

[20] Yang M., Hu M., Zhang Y., Jia S., Sun X., Zhao W., Ge M., Dong B. (2022). Sarcopenic Obesity Is Associated with Frailty among Community-Dwelling Older Adults: Findings from the WCHAT Study. BMC Geriatr..

[21] Damanti S., Citterio L., Zagato L., Brioni E., Magnaghi C., Simonini M., De Lorenzo R., Ruggiero M., Santoro S., Senini E. (2024). Sarcopenic Obesity and Pre-Sarcopenia Contribute to Frailty in Community-Dwelling Italian Older People: Data from the FRASNET Study. BMC Geriatr..

[22] Baumgartner R.N., Wayne S.J., Waters D.L., Janssen I., Gallagher D., Morley J.E. (2004). Sarcopenic Obesity Predicts Instrumental Activities of Daily Living Disability in the Elderly. Obes. Res..

[23] Silay K., Selvi Oztorun H. (2025). Sarcopenic Obesity Is Linked to Worse Clinical Outcomes than Sarcopenia or Obesity Alone in Hospitalized Older Adults. BMC Geriatr..

[24] Khatoon B S., Saravanan D., Ganamurali N., Sabarathinam S. (2023). A Narrative Review on the Impact of Sarcopenic Obesity and Its Psychological Consequence in Quality of Life. Diabetes Metab. Syndr. Clin. Res. Rev..

[25] Cho Y., Shin S.Y., Shin M.J. (2015). Sarcopenic Obesity Is Associated with Lower Indicators of Psychological Health and Quality of Life in Koreans. Nutr. Res..

[26] Hong S.H., Choi K.M. (2020). Sarcopenic Obesity, Insulin Resistance, and Their Implications in Cardiovascular and Metabolic Consequences. Int. J. Mol. Sci..

[27] Wang M., Tan Y., Shi Y., Wang X., Liao Z., Wei P. (2020). Diabetes and Sarcopenic Obesity: Pathogenesis, Diagnosis, and Treatments. Front. Endocrinol..

[28] Lou Y., Xie Y., Jiang Q., Huang S., Wang X., Wang L., Wang H., Cao S. (2025). The Effect of Sarcopenic Obesity on Glycaemic Status Based on Fasting Plasma Glucose and Glycated Haemoglobin: A Prospective Cohort Study. Diabetes Obes. Metab..

[29] Filis P., Papagiannopoulos C.K., Markozannes G., Chalitsios C.V., Zerdes I., Valachis A., Papandreou C., Christakoudi S., Tsilidis K.K. (2025). Associations of Sarcopenia, Sarcopenia Components and Sarcopenic Obesity with Cancer Incidence: A Prospective Cohort Study of 414,094 Participants in UK Biobank. Int. J. Cancer.

[30] Wang Y., Liu X., Zhang X., Fan P., Zong Y., Feng X., Huang L. (2025). Impact of Sarcopenic Obesity on Postoperative Complications and Long-Term Survival in Patients with Gastric Cancer. Eur. J. Surg. Oncol..

[31] Liu C., Liu T., Deng L., Zhang Q., Song M., Shi J., Liu C., Xie H., Chen Y., Lin S. (2024). Sarcopenic Obesity and Outcomes for Patients with Cancer. JAMA Netw. Open.

[32] Kim D., Lee J., Park R., Oh C.M., Moon S. (2024). Association of Low Muscle Mass and Obesity with Increased All-Cause and Cardiovascular Disease Mortality in US Adults. J. Cachexia Sarcopenia Muscle.

[33] Farmer R.E., Mathur R., Schmidt A.F., Bhaskaran K., Fatemifar G., Eastwood S.V., Finan C., Denaxas S., Smeeth L., Chaturvedi N. (2019). Associations Between Measures of Sarcopenic Obesity and Risk of Cardiovascular Disease and Mortality: A Cohort Study and Mendelian Randomization Analysis Using the UK Biobank. J. Am. Heart Assoc..

[34] Conte C., Hall K.D., Klein S. (2024). Is Weight Loss-Induced Muscle Mass Loss Clinically Relevant?. JAMA.

[35] Sakuma K., Yamaguchi A. (2013). Sarcopenic Obesity and Endocrinal Adaptation with Age. Int. J. Endocrinol..

[36] Huang L.T., Wang J.H. (2021). The Therapeutic Intervention of Sex Steroid Hormones for Sarcopenia. Front. Med..

[37] Sørensen M.B., Rosenfalck A.M., Højgaard L., Ottesen B. (2001). Obesity and Sarcopenia after Menopause Are Reversed by Sex Hormone Replacement Therapy. Obes. Res..

[38] Bian A., Ma Y., Zhou X., Guo Y., Wang W., Zhang Y., Wang X. (2020). Association between Sarcopenia and Levels of Growth Hormone and Insulin-like Growth Factor-1 in the Elderly. BMC Musculoskelet. Disord..

[39] Xu Q., Bu F., Song Z.T., Li K., Fang C., Luo Y., Zhang L., Pei Y.F. (2025). Association of Serum 25-Hydroxyvitamin D with Sarcopenic Obesity Risk: A Longitudinal Observational Study from the UK Biobank. Obesity.

[40] Kalinkovich A., Livshits G. (2017). Sarcopenic Obesity or Obese Sarcopenia: A Cross Talk between Age-Associated Adipose Tissue and Skeletal Muscle Inflammation as a Main Mechanism of the Pathogenesis. Ageing Res. Rev..

[41] Shulman G.I. (2000). Cellular Mechanisms of Insulin Resistance. J. Clin. Investig..

[42] Aoi W. (2021). Myokines: A Potential Key Factor in Development, Treatment, and Biomarker of Sarcopenia. Sarcopenia: Molecular Mechanism and Treatment Strategies.

[43] Kim T.N., Park M.S., Ryu J.Y., Choi H.Y., Hong H.C., Yoo H.J., Kang H.J., Song W., Park S.W., Baik S.H. (2014). Impact of Visceral Fat on Skeletal Muscle Mass and Vice Versa in a Prospective Cohort Study: The Korean Sarcopenic Obesity Study (KSOS). PLoS ONE.

[44] Wan M., Gray-Gaillard E.F., Elisseeff J.H. (2021). Cellular Senescence in Musculoskeletal Homeostasis, Diseases, and Regeneration. Bone Res..

[45] He Y., Xie W., Li H., Jin H., Zhang Y., Li Y. (2022). Cellular Senescence in Sarcopenia: Possible Mechanisms and Therapeutic Potential. Front. Cell Dev. Biol..

[46] de Marco Castro E., Murphy C.H., Roche H.M. (2021). Targeting the Gut Microbiota to Improve Dietary Protein Efficacy to Mitigate Sarcopenia. Front. Nutr..

[47] Fernández-Martínez J., Ramírez-Casas Y., Yang Y., Aranda-Martínez P., Martínez-Ruiz L., Escames G., Acuña-Castroviejo D. (2023). From Chronodisruption to Sarcopenia: The Therapeutic Potential of Melatonin. Biomolecules.

[48] Gandham A., Zengin A., Bonham M.P., Brennan-Olsen S.L., Aitken D., Winzenberg T.M., Ebeling P.R., Jones G., Scott D. (2021). Associations between Socioeconomic Status and Obesity, Sarcopenia, and Sarcopenic Obesity in Community-Dwelling Older Adults: The Tasmanian Older Adult Cohort Study. Exp. Gerontol..

[49] Batsis J.A., Mackenzie T.A., Lopez-Jimenez F., Bartels S.J. (2015). Sarcopenia, Sarcopenic Obesity, and Functional Impairments in Older Adults: National Health and Nutrition Examination Surveys 1999–2004. Nutr. Res..

[50] Donini L.M., Busetto L., Bischoff S.C., Cederholm T., Ballesteros-Pomar M.D., Batsis J.A., Bauer J.M., Boirie Y., Cruz-Jentoft A.J., Dicker D. (2022). Definition and Diagnostic Criteria for Sarcopenic Obesity: ESPEN and EASO Consensus Statement. Obes. Facts.

[51] Chen T.P., Kao H.H., Ogawa W., Arai H., Tahapary D.L., Assantachai P., Tham K.W., Chan D.C., Yuen M.M.A., Appannah G. (2025). The Asia–Oceania Consensus: Definitions and Diagnostic Criteria for Sarcopenic Obesity. Obes. Res. Clin. Pract..

[52] Woo J., Leung J., Morley J.E. (2014). Validating the SARC-F: A Suitable Community Screening Tool for Sarcopenia?. J. Am. Med. Dir. Assoc..

[53] Ha Y.C., Won C.W., Kim M., Chun K.J., Yoo J. (2020). Il SARC-F as a Useful Tool for Screening Sarcopenia in Elderly Patients with Hip Fractures. J. Nutr. Health Aging.

[54] Rubino F., Cummings D.E., Eckel R.H., Cohen R.V., Wilding J.P.H., Brown W.A., Stanford F.C., Batterham R.L., Farooqi I.S., Farpour-Lambert N.J. (2025). Definition and Diagnostic Criteria of Clinical Obesity. Lancet Diabetes Endocrinol..

[55] Ida S., Kaneko R., Murata K. (2018). SARC-F for Screening of Sarcopenia Among Older Adults: A Meta-Analysis of Screening Test Accuracy. J. Am. Med. Dir. Assoc..

[56] Cesari M., Fielding R.A., Pahor M., Goodpaster B., Hellerstein M., van Kan G.A., Anker S.D., Rutkove S., Vrijbloed J.W., Isaac M. (2012). Biomarkers of Sarcopenia in Clinical Trials-Recommendations from the International Working Group on Sarcopenia. J. Cachexia Sarcopenia Muscle.

[57] Konečná M., Poráčová J., Sedlák V., Gaľová J., Babejová A., Zahatňanská M., Kimáková T., Nagy M., Bernátová R., Bernát M. (2023). Use of Bioimpedance in Prevention of Sarcopenia in the Elderly. Cent. Eur. J. Public Health.

[58] Wareham N.J. (2022). Personalised Prevention of Type 2 Diabetes. Diabetologia.

[59] Caturano A., Amaro A., Berra C.C., Conte C. (2025). Sarcopenic Obesity and Weight Loss-Induced Muscle Mass Loss. Curr. Opin. Clin. Nutr. Metab. Care.

[60] Volkert D., Beck A.M., Cederholm T., Cruz-Jentoft A., Hooper L., Kiesswetter E., Maggio M., Raynaud-Simon A., Sieber C., Sobotka L. (2022). ESPEN Practical Guideline: Clinical Nutrition and Hydration in Geriatrics. Clin. Nutr..

[61] Trouwborst I., Verreijen A., Memelink R., Massanet P., Boirie Y., Weijs P., Tieland M. (2018). Exercise and Nutrition Strategies to Counteract Sarcopenic Obesity. Nutrients.

[62] Volpe S.L., Sukumar D., Milliron B.J. (2016). Obesity Prevention in Older Adults. Curr. Obes. Rep..

[63] Irvin L., Madden L.A., Marshall P., Vince R.V. (2023). Digital Health Solutions for Weight Loss and Obesity: A Narrative Review. Nutrients.

[64] Bae J.H., Seo J.W., Li X., Ahn S.Y., Sung Y., Kim D.Y. (2024). Neural Network Model for Prediction of Possible Sarcopenic Obesity Using Korean National Fitness Award Data (2010–2023). Sci. Rep..

[65] Zambon Azevedo V., Ponnaiah M., Bel Lassen P., Ratziu V., Oppert J.M. (2022). A Diagnostic Proposal for Sarcopenic Obesity in Adults Based on Body Composition Phenotypes. Clin. Nutr. ESPEN.

[66] Lian R., Tang H., Chen Z., Chen X., Luo S., Jiang W., Jiang J., Yang M. (2025). Development and Multi-Center Cross-Setting Validation of an Explainable Prediction Model for Sarcopenic Obesity: A Machine Learning Approach Based on Readily Available Clinical Features. Aging Clin. Exp. Res..

[67] Duff O.M., Walsh D.M.J., Furlong B.A., O’Connor N.E., Moran K.A., Woods C.B. (2017). Behavior Change Techniques in Physical Activity EHealth Interventions for People With Cardiovascular Disease: Systematic Review. J. Med. Internet Res..

[68] Muellmann S., Forberger S., Möllers T., Bröring E., Zeeb H., Pischke C.R. (2018). Effectiveness of EHealth Interventions for the Promotion of Physical Activity in Older Adults: A Systematic Review. Prev. Med..

[69] Duan Y., Shang B., Liang W., Du G., Yang M., Rhodes R.E. (2021). Effects of EHealth-Based Multiple Health Behavior Change Interventions on Physical Activity, Healthy Diet, and Weight in People with Noncommunicable Diseases: Systematic Review and Meta-Analysis. J. Med. Internet Res..

[70] Schoufour J.D., Tieland M., Barazzoni R., Ben Allouch S., van der Bie J., Boirie Y., Cruz-Jentoft A.J., Eglseer D., Topinková E., Visser B. (2021). The Relevance of Diet, Physical Activity, Exercise, and Persuasive Technology in the Prevention and Treatment of Sarcopenic Obesity in Older Adults. Front. Nutr..

[71] Bailey D.P., Harper J.H., Kilbride C., McGowan L.J., Victor C., Brierley M.L., Chater A.M. (2024). The Frail-LESS (LEss Sitting and Sarcopenia in Frail Older Adults) Remote Intervention to Improve Sarcopenia and Maintain Independent Living via Reductions in Sedentary Behaviour: Findings from a Randomised Controlled Feasibility Trial. BMC Geriatr..

[72] Shi B., Li G., Wu S., Ge H., Zhang X., Chen S., Pan Y., He Q. (2024). Assessing the Effectiveness of EHealth Interventions to Manage Multiple Lifestyle Risk Behaviors Among Older Adults: Systematic Review and Meta-Analysis. J. Med. Internet Res..

[73] Lemos T., Gallagher D. (2017). Current Body Composition Measurement Techniques. Curr. Opin. Endocrinol. Diabetes Obes..

[74] Johnson Stoklossa C.A., Forhan M., Padwal R.S., Gonzalez M.C., Prado C.M. (2016). Practical Considerations for Body Composition Assessment of Adults with Class II/III Obesity Using Bioelectrical Impedance Analysis or Dual-Energy X-Ray Absorptiometry. Curr. Obes. Rep..

[75] Beausejour J.P., Knowles K.S., Wilson A.T., Mangum L.C., Hill E.C., Hanney W.J., Wells A.J., Fukuda D.H., Stout J., Stock M.S. (2024). Innovations in the Assessment of Skeletal Muscle Health: A Glimpse into the Future. Int. J. Sports Med..

[76] Qiao C., Rolfe E.D.L., Mak E., Sengupta A., Powell R., Watson L.P.E., Heymsfield S.B., Shepherd J.A., Wareham N., Brage S. (2024). Prediction of Total and Regional Body Composition from 3D Body Shape. Npj Digit. Med..

[77] Florez C.M., Rodriguez C., Siedler M.R., Tinoco E., Tinsley G.M. (2024). Body Composition Estimation from Mobile Phone Three-Dimensional Imaging: Evaluation of the USA Army One-Site Method. Br. J. Nutr..

[78] Graybeal A.J., Brandner C.F., Tinsley G.M. (2022). Visual Body Composition Assessment Methods: A 4-Compartment Model Comparison of Smartphone-Based Artificial Intelligence for Body Composition Estimation in Healthy Adults. Clin. Nutr..

[79] Graybeal A.J., Swafford S.H., Compton A.T., Renna M.E., Thorsen T., Stavres J. (2025). Predicting Bone Mineral Content from Smartphone Digital Anthropometrics: Evaluation of an Existing Application and the Development of New Prediction Models. J. Clin. Densitom..

[80] Scott D., Chandrasekara S.D., Laslett L.L., Cicuttini F., Ebeling P.R., Jones G. (2016). Associations of Sarcopenic Obesity and Dynapenic Obesity with Bone Mineral Density and Incident Fractures Over 5–10 Years in Community-Dwelling Older Adults. Calcif. Tissue Int..

[81] Bennett J.P., Liu Y.E., Kelly N.N., Quon B.K., Wong M.C., McCarthy C., Heymsfield S.B., Shepherd J.A. (2022). Next-Generation Smart Watches to Estimate Whole-Body Composition Using Bioimpedance Analysis: Accuracy and Precision in a Diverse, Multiethnic Sample. Am. J. Clin. Nutr..

[82] Thomas D.M., Crofford I., Scudder J., Oletti B., Deb A., Heymsfield S.B. (2025). Updates on Methods for Body Composition Analysis: Implications for Clinical Practice. Curr. Obes. Rep..

[83] Brandner C.F., Tinsley G.M., Graybeal A.J. (2023). Smartwatch-Based Bioimpedance Analysis for Body Composition Estimation: Precision and Agreement with a 4-Compartment Model. Appl. Physiol. Nutr. Metab..

[84] Ruiz-Cárdenas J.D., Montemurro A., del Mar Martínez-García M., Rodríguez-Juan J.J. (2023). Sit-to-Stand Video Analysis-Based App for Diagnosing Sarcopenia and Its Relationship with Health-Related Risk Factors and Frailty in Community-Dwelling Older Adults: Diagnostic Accuracy Study. J. Med. Internet Res..

[85] Vavasour G., Giggins O.M., Doyle J., Kelly D. (2021). How Wearable Sensors Have Been Utilised to Evaluate Frailty in Older Adults: A Systematic Review. J. Neuroeng. Rehabil..

[86] Zhang Y., Morita M., Hirano T., Doi K., Han X., Matsunaga K., Jiang Z. (2024). A Novel Method for Identifying Frailty and Quantifying Muscle Strength Using the Six-Minute Walking Test. Sensors.

[87] Connolly K., Cunningham C., Murphy N., Romero-Ortuno R., Horgan F. (2021). Using Accelerometers in the Assessment of Sarcopenia in Older Adults Attending a Day Hospital Service in Ireland. J. Frailty Sarcopenia Falls.

[88] Shin J., Kweon H.J., Choi J. (2025). Assessment of Gait Parameters Using Wearable Sensors and Their Association with Muscle Mass, Strength, and Physical Performance in Korean Older Adults: Cross-Sectional Study. JMIR Form. Res..

[89] Aznar-Gimeno R., Perez-Lasierra J.L., Pérez-Lázaro P., Bosque-López I., Azpíroz-Puente M., Salvo-Ibáñez P., Morita-Hernandez M., Hernández-Ruiz A.C., Gómez-Bernal A., Rodrigalvarez-Chamarro M.d.l.V. (2024). Gait-Based AI Models for Detecting Sarcopenia and Cognitive Decline Using Sensor Fusion. Diagnostics.

[90] Perez-Lasierra J.L., Azpíroz-Puente M., Alfaro-Santafé J.V., Almenar-Arasanz A.J., Alfaro-Santafé J., Gómez-Bernal A. (2024). Sarcopenia Screening Based on the Assessment of Gait with Inertial Measurement Units: A Systematic Review. BMC Geriatr..

[91] Han S., Xiao Q., Liang Y., Chen Y., Yan F., Chen H., Yue J., Tian X., Xiong Y. (2024). Using Flexible-Printed Piezoelectric Sensor Arrays to Measure Plantar Pressure during Walking for Sarcopenia Screening. Sensors.

[92] Huang Z., Ou Q., Li D., Feng Y., Cai L., Hu Y., Chu H. (2024). Wearable Fabric System for Sarcopenia Detection. Biosensors.

[93] Rescio G., Sciurti E., Giampetruzzi L., Carluccio A.M., Francioso L., Leone A. (2025). Preliminary Study on Wearable Smart Socks with Hydrogel Electrodes for Surface Electromyography-Based Muscle Activity Assessment. Sensors.

[94] Shin J., Song K., Kim S.W., Choi S., Lee H., Kim I.S., Im S., Baek M.S. (2025). A Wearable Approach for Sarcopenia Diagnosis Using Stimulated Muscle Contraction Signal. Biomed. Eng. Lett..

[95] Liarakos A.L., Panagiotou G., Chondronikola M., Wilmot E.G. (2025). Continuous Glucose Monitoring in People at High Risk of Diabetes and Dysglycaemia: Transforming Early Risk Detection and Personalised Care. Life.

[96] Metwally A.A., Perelman D., Park H., Wu Y., Jha A., Sharp S., Celli A., Ayhan E., Abbasi F., Gloyn A.L. (2025). Prediction of Metabolic Subphenotypes of Type 2 Diabetes via Continuous Glucose Monitoring and Machine Learning. Nat. Biomed. Eng..

[97] Park H., Metwally A.A., Delfarah A., Wu Y., Perelman D., Mayer C., McGinity C., Rodgar M., Celli A., McLaughlin T. (2025). High-Resolution Lifestyle Profiling and Metabolic Subphenotypes of Type 2 Diabetes. npj Digit. Med..

[98] Starkoff B.E., Nickerson B.S. (2024). Emergence of Imaging Technology beyond the Clinical Setting: Utilization of Mobile Health Tools for at-Home Testing. Nutr. Clin. Pract..

[99] Watanabe K., Wilmanski T., Diener C., Earls J.C., Zimmer A., Lincoln B., Hadlock J.J., Lovejoy J.C., Gibbons S.M., Magis A.T. (2023). Multiomic Signatures of Body Mass Index Identify Heterogeneous Health Phenotypes and Responses to a Lifestyle Intervention. Nat. Med..

[100] Sacoto D., Hurtado M.D., Acosta A. (2022). Precision Medicine and Obesity. Handb. Exp. Pharmacol..

[101] Woldemariam S., Dorner T.E., Wiesinger T., Stein K.V. (2023). Multi-Omics Approaches for Precision Obesity Management: Potentials and Limitations of Omics in Precision Prevention, Treatment and Risk Reduction of Obesity. Wien. Klin. Wochenschr.

[102] Lynch D.H., Spangler H.B., Franz J.R., Krupenevich R.L., Kim H., Nissman D., Zhang J., Li Y.Y., Sumner S., Batsis J.A. (2022). Multimodal Diagnostic Approaches to Advance Precision Medicine in Sarcopenia and Frailty. Nutrients.

[103] Liu J.C., Dong S.S., Shen H., Yang D.Y., Chen B.-B., Ma X.Y., Peng Y.R., Xiao H.M., Deng H.W. (2022). Multi-Omics Research in Sarcopenia: Current Progress and Future Prospects. Ageing Res. Rev..

[104] Wei S., Nguyen T.T., Zhang Y., Ryu D., Gariani K. (2023). Sarcopenic Obesity: Epidemiology, Pathophysiology, Cardiovascular Disease, Mortality, and Management. Front. Endocrinol..

[105] Ran S., Jiang Z.X., He X., Liu Y., Zhang Y.X., Zhang L., Pei Y.F., Zhang M., Hai R., Gu G.S. (2020). Replication of FTO Gene Associated with Lean Mass in a Meta-Analysis of Genome-Wide Association Studies. Sci. Rep..

[106] Semenova E.A., Pranckevičienė E., Bondareva E.A., Gabdrakhmanova L.J., Ahmetov I.I. (2023). Identification and Characterization of Genomic Predictors of Sarcopenia and Sarcopenic Obesity Using UK Biobank Data. Nutrients.

[107] Xu Q., Zhao Q.G., Ma X.L., Yan S.S., Han B.X., Song Z.T., Bu F., Li K., Zhang L., Pei Y.F. (2024). Exome-Wide Sequencing Study Identified Genetic Variants Associated with Sarcopenic Obesity. J. Gerontol. Ser. A.

[108] Yang Y., Fan J., Xu H., Fan L., Deng L., Li J., Li D., Li H., Zhang F., Zhao R.C. (2021). Long Noncoding RNA LYPLAL1-AS1 Regulates Adipogenic Differentiation of Human Mesenchymal Stem Cells by Targeting Desmoplakin and Inhibiting the Wnt/β-Catenin Pathway. Cell Death Discov..

[109] Ikeda Y., Kawamura R., Takata Y., Tabara Y., Maruyama K., Takakado M., Hadate T., Ohashi J., Saito I., Ogawa Y. (2023). Resistin G–A Haplotype at SNP-420/-358 Is Associated with the Latent Sarcopenic Obesity Index in the Toon Genome Study. J. Diabetes Investig..

[110] Burton M.A., Antoun E., Garratt E.S., Westbury L., Baczynska A., Dennison E.M., Harvey N.C., Cooper C., Patel H.P., Godfrey K.M. (2023). Adiposity Is Associated with Widespread Transcriptional Changes and Downregulation of Longevity Pathways in Aged Skeletal Muscle. J. Cachexia Sarcopenia Muscle.

[111] Tarum J., Ball G., Gustafsson T., Altun M., Santos L. (2024). Artificial Neural Network Inference Analysis Identified Novel Genes and Gene Interactions Associated with Skeletal Muscle Aging. J. Cachexia Sarcopenia Muscle.

[112] Shu H., Huang Y., Zhang W., Ling L., Hua Y., Xiong Z. (2023). An Integrated Study of Hormone-Related Sarcopenia for Modeling and Comparative Transcriptome in Rats. Front. Endocrinol..

[113] Chen J., Xu J., Gou L., Zhu Y., Zhong W., Guo H., Du Y. (2024). Integrating Transcriptomic and Proteomic Data for a Comprehensive Molecular Perspective on the Association between Sarcopenia and Osteoporosis. Arch. Gerontol. Geriatr..

[114] Yu R., Miao L., Wang J., Wang D., Liu Y., Wang L., Wang R. (2025). Integrated Transcriptomics Unveils Mitochondrial Oxidative Phosphorylation Dysfunction as a Shared Mechanism in Sarcopenia and Obesity. Sci. Rep..

[115] Huemer M.T., Bauer A., Petrera A., Scholz M., Hauck S.M., Drey M., Peters A., Thorand B. (2021). Proteomic Profiling of Low Muscle and High Fat Mass: A Machine Learning Approach in the KORA S4/FF4 Study. J. Cachexia Sarcopenia Muscle.

[116] Hinkley J.M., Cornnell H.H., Standley R.A., Chen E.Y., Narain N.R., Greenwood B.P., Bussberg V., Tolstikov V.V., Kiebish M.A., Yi F. (2020). Older Adults with Sarcopenia Have Distinct Skeletal Muscle Phosphodiester, Phosphocreatine, and Phospholipid Profiles. Aging Cell.

[117] Yang Q., Zhang Z., He P., Mao X., Jing X., Hu Y., Jing L. (2024). LC/MS-Based Untargeted Lipidomics Reveals Lipid Signatures of Sarcopenia. Int. J. Mol. Sci..

[118] Han P., Yuan C., Chen X., Hu Y., Hu X., Xu Z., Guo Q. (2024). Metabolic Signatures and Potential Biomarkers of Sarcopenia in Suburb-Dwelling Older Chinese: Based on Untargeted GC–MS and LC–MS. Skelet. Muscle.

